# Vocabulary Knowledge and Braille Reading Comprehension in Chinese Students with Blindness: Contributions of Compounding Awareness and Lexical Inferencing

**DOI:** 10.3390/bs16071100

**Published:** 2026-07-02

**Authors:** Zhilin Wu, Mengyu Tian, Qi Wang, Kaixin Li, Tong Lin, Yuexin Zhang

**Affiliations:** 1 Faculty of Education, East China Normal University, Shanghai 200062, China; 15378806988@163.com; 2 College of Education for the Future, Beijing Normal University, Zhuhai 519087, China; 3 Shenzhen Futian Special Education Resources Center, Shenzhen 518031, China; m13121516169@163.com; 4 Faculty of Arts and Sciences, Beijing Normal University, Zhuhai 519087, China; 202411079520@mail.bnu.edu.cn (K.L.); 202311079208@mail.bnu.edu.cn (T.L.); 5 Faculty of Education, Beijing Normal University, Beijing 100875, China

**Keywords:** students with blindness, compounding awareness, lexical inferencing, vocabulary knowledge, Chinese Braille reading comprehension

## Abstract

Although lexical inferencing, defined as morpheme-based inference of unfamiliar compound word meanings, is a key contributor to reading development in sighted children, its contribution to Braille reading comprehension among students with blindness remains underexplored, representing an important gap in research on Braille reading development. This study had two aims. First, it aimed to characterize lexical inferencing in Chinese primary school students with blindness by comparing their performance with that of sighted peers. Second, it aimed to examine the associations of lexical inferencing and compounding awareness with vocabulary knowledge and Braille reading comprehension among students with blindness, and to determine whether these associations differed between middle- and upper-grade students. The study included valid data from 101 students with blindness in Grades 3–6 and 142 sighted students in Grades 2–6. Students with blindness completed measures of compounding awareness, lexical inferencing, vocabulary knowledge, and Braille reading comprehension, whereas sighted students completed only the lexical inferencing task. Hierarchical regression analyses were conducted on a sample of students with blindness to examine the relationships among the four skills. Results showed that (1) students with blindness performed lower than sighted peers on lexical inferencing despite grade-related improvement; (2) lexical inferencing significantly predicted both vocabulary knowledge and Braille reading comprehension in middle- and upper-grade students, whereas compounding awareness significantly predicted lexical inferencing; (3) the relative roles of compounding awareness and lexical inferencing differed by grade group. In middle-grade students, both compounding awareness and lexical inferencing contributed to vocabulary knowledge and Braille reading comprehension, with vocabulary knowledge also predicting reading comprehension. In upper-grade students, lexical inferencing remained a significant predictor of both vocabulary knowledge and Braille reading comprehension, whereas compounding awareness no longer directly predicted either outcome. These findings indicate a developmental shift in which compounding awareness is more influential in earlier stages, whereas lexical inferencing plays a more important role in vocabulary and text-level comprehension in later stages.

## 1. Introduction

Reading comprehension requires the integration of multiple linguistic and cognitive processes, including word recognition, vocabulary knowledge, and the ability to infer meaning beyond explicitly stated information (Lundberg, 2002; Chen et al., 2019). Although these processes have been extensively examined in sighted readers, how they function in Braille reading is less well understood, particularly in non-alphabetic languages.

Braille is a tactile writing system in which readers move their fingers across patterns of raised dots, with each character represented by a six-dot cell arranged in two columns and three rows. From this perspective, Braille reading should be understood at two related levels: as a tactile encoding system and as a linguistic interface between tactile symbols and a particular written language. On the one hand, tactile input is typically slower and more sequential than visual print processing. On the other hand, Braille is also a linguistic interface, with the mapping between Braille symbols and language varying according to the writing system being represented. This distinction is important because findings from Western Braille literacy research provide a useful foundation for understanding tactile reading, but their implications may differ across alphabetic and logographic writing systems such as Chinese.

In alphabetic Braille systems such as English Braille, many Braille symbols are designated to represent the letters of the Roman alphabet (e.g., “
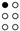
” represents “a,” “
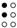
” represents “b,” and “
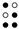
” represents “z”). Although English Braille also includes contractions and other system-specific conventions, its basic mapping preserves much of the letter-based structure of alphabetic print. As a result, Braille readers can often access phonological and lexical representations through symbol-to-letter or symbol-to-word mappings that are broadly comparable to those involved in alphabetic print reading. In contrast, Chinese Braille adopts a fundamentally different encoding principle. Many Chinese characters provide both phonological and semantic cues. For example, the character “河” (/hé/, river) contains the semantic radical “氵,” which signals a meaning related to water. In contrast, Chinese Braille represents spoken syllables using Pinyin, a Romanization system for Mandarin Chinese that encodes only pronunciation. Thus, the word “河流” (river) is represented through its syllabic pronunciation rather than through visually meaningful character forms. As a result, Chinese Braille provides substantially fewer direct form–meaning cues than written Chinese characters. This challenge is further complicated by the high prevalence of homophony in Chinese. Many different Chinese characters share identical pronunciations despite having unrelated meanings. For example, “事” (matter), “市” (city), “试” (test), and “是” (to be) are all pronounced /shì/. Because Chinese Braille encodes pronunciation rather than character form, these distinctions cannot be directly recovered from Braille orthography alone. Readers must therefore rely more heavily on morphological structure, lexical knowledge, and inferential processes to identify intended meanings during reading. Consequently, compounding awareness, vocabulary knowledge, and lexical inferencing may be particularly important for Chinese Braille reading comprehension.

Building on these characteristics of Chinese Braille, one reading-related skill that deserves attention is compounding awareness. Compounding awareness refers to the understanding of how morphemes can be combined sensibly in Chinese (Liu & McBride-Chang, 2010; Xia et al., 2023). Because Chinese has many compound words, readers often need to use the meanings of constituent morphemes and structural relations among them to construct word meaning. In Chinese Braille reading, compounding awareness is important because readers often need to construct word meaning without direct access to the character-form cues available in printed Chinese.

Vocabulary knowledge is another central component of reading comprehension. It refers to the knowledge of word meanings that individuals draw upon to comprehend others’ speech, express their own ideas, and interpret written texts (Moats, 2005). Vocabulary knowledge is commonly divided into breadth and depth. Vocabulary breadth reflects the size of an individual’s lexical repertoire, whereas vocabulary depth captures the extent to which individuals understand word meanings and their appropriate use in context (Ouellette, 2006; Xie et al., 2023). In this study, vocabulary knowledge was operationalized as vocabulary depth, assessed through students’ ability to define words. This measure was chosen because precise semantic knowledge is more directly relevant to text comprehension than simple word familiarity.

Previous studies have compared students with blindness and sighted students in terms of compounding awareness, vocabulary knowledge (Xie et al., 2023; Chen et al., 2023), and shown that compounding awareness and vocabulary knowledge are associated with Chinese Braille reading. Compounding awareness has been found to predict Braille reading comprehension, particularly among students in Grades 3–4 (Xia et al., 2023). It has also been identified as a unique predictor of vocabulary knowledge, explaining 29% of the variance in vocabulary knowledge among students in Grades 1–3 and 10% among students in Grades 4–6 (Xie et al., 2023). In addition, vocabulary knowledge may mediate the relationship between morphological awareness and Braille reading comprehension among students in Grades 3–6 (Chen et al., 2019). These studies establish compounding awareness and vocabulary knowledge as important correlates of Chinese Braille reading. However, they focus mainly on students’ existing morphological and lexical knowledge. Less is known about how students use this knowledge to infer the meanings of unfamiliar words, especially as Braille texts become more complex in the upper primary grades.

This question points to the role of lexical inferencing. Lexical inferencing is generally defined as the ability to make informed guesses about the meanings of unknown or unfamiliar words by drawing on available linguistic cues, world knowledge, contextual awareness, and relevant linguistic knowledge (Haastrup, 1991). Lexical inferencing can draw on different sources of information, including contextual cues and morphemic cues (Baumann et al., 2003; Nagy & Scott, 2000; Wysocki & Jenkins, 1987; H. Zhang et al., 2022). In the present study, lexical inferencing was operationalized as morpheme-based lexical inferencing of unfamiliar compound word meanings. Levesque et al. (2019) referred to this process as “morphological analysis.” It involves employing constituent morphemes to derive the meanings of unfamiliar morphologically complex words. Compounding awareness and lexical inferencing are closely related but conceptually distinct. Both lexical inference and metalinguistic awareness involve the processing of word-internal structures (Koda, 2005). However, at a conceptual level, lexical inferencing is grounded in the competence to identify structural properties from abstract linguistic forms rather than being a by-product of metalinguistic capabilities (Cheng & Zhang, 2023). Compounding awareness refers to a metalinguistic ability that reflects individuals’ explicit sensitivity to the internal morphological structure of compound words, typically assessed in decontextualized tasks requiring judgment or manipulation of word components. Unlike compounding awareness, which reflects knowledge of morphological structure itself, in the present study, lexical inferencing, conceptualized as morphological analysis, refers to the online process by which individual morphemic elements are accessed and integrated to interpret the meanings of unfamiliar morphologically complex words (Levesque et al., 2019). Examining both variables therefore makes it possible to distinguish between morphological knowledge itself and the application of that knowledge during unfamiliar-word meaning inference.

In sighted Chinese children, lexical inferencing has been shown to predict text-level reading comprehension after controlling for phonological awareness, morphological awareness, and orthographic awareness, with its contribution increasing across Grades 3–5 (Cheng & Zhang, 2023). It also predicts vocabulary growth, and compounding awareness may contribute to vocabulary acquisition indirectly through lexical inferencing (H. Zhang, 2015). These findings suggest that lexical inferencing may provide a pathway through which children use morphological knowledge to derive the meanings of unfamiliar words, thereby predicting both vocabulary growth and reading comprehension.

Lexical inferencing is also relevant to vocabulary knowledge. When readers encounter unfamiliar words, they can use morphemic cues in the lexical form and integrate them with prior knowledge to infer meaning (Baumann et al., 2003). Repeated encounters with such words across contexts can further refine semantic representations and strengthen knowledge of their relations to other lexical items (Ricketts et al., 2011). Thus, lexical inferencing is not only a strategy for resolving immediate comprehension difficulty but also a potential route through which unfamiliar words become incorporated into the mental lexicon. These issues are especially relevant to Chinese Braille reading, yet lexical inferencing has rarely been examined in students with blindness. Existing studies have mainly focused on morphological awareness, phonological awareness, vocabulary knowledge, and reading comprehension (Chen et al., 2019, 2023; Xia et al., 2023; Xie et al., 2023). It therefore remains unclear whether lexical inferencing contributes to vocabulary knowledge and Braille reading comprehension beyond compounding awareness, and whether its role differs between middle-grade students (Grades 3–4) and upper-grade students (Grades 5–6).

The present study examined how compounding awareness, lexical inferencing, and vocabulary knowledge are associated with Braille reading comprehension in Chinese students with blindness. We analyzed these relations separately in middle-grade students (Grades 3–4) and upper-grade students (Grades 5–6), because the contribution of these skills may change as students’ Braille reading becomes more fluent and text demands increase. Sighted students were included as a sighted comparison group. Specifically, the study addressed four research questions. First, how does lexical inferencing performance in Chinese students with blindness compare with that of sighted students? Second, does lexical inferencing predict vocabulary knowledge and Braille reading comprehension in middle-grade students (Grades 3–4) and upper-grade students (Grades 5–6)? Third, does compounding awareness predict lexical inferencing in these two grade groups? Fourth, between compounding awareness and lexical inferencing, which shows the more salient predictive role in vocabulary knowledge and Braille reading comprehension in middle-grade and upper-grade students?

## 2. Materials and Methods

### 2.1. Participants

Following approval from the Research Ethics Committee of Beijing Normal University (see Section 2.3 and the Institutional Review Board Statement for details on ethical procedures), this study employed a cluster random sampling strategy across eastern, central, and western regions of China, including Beijing, Qingdao, Yantai, Tai’an, Nanjing, Taiyuan, and Yinchuan. One special education school was selected from each city (seven schools in total), yielding a sample of 107 blind students in Grades 3–6. These students completed assessments of compounding awareness, lexical inferencing, vocabulary knowledge, and Braille reading comprehension. No participants with blindness had additional disabilities beyond visual impairment and demonstrated functional proficiency in Braille. All participants with blindness were legally classified as blind and were Braille users.

A regular primary school in Beijing served as the control site. One class was randomly selected from each grade level (Grades 2–6), resulting in 148 sighted students, all native speakers of Chinese.

The inclusion of blind students from Grade 3 onward and sighted students from Grade 2 onward was guided by developmental and curricular considerations. According to the Compulsory Education Chinese Language Curriculum Standards for Schools for the Blind (Ministry of Education of the People’s Republic of China, 2016a, 2016b, 2016c), students in Grades 1–2 primarily acquire foundational knowledge of Braille dot patterns and have not yet achieved fluent tactile reading. From Grade 3, they are expected to read and write Braille proficiently. Similarly, the Compulsory Education Chinese Language Curriculum Standards (Ministry of Education of the People’s Republic of China, 2022) indicate that Grade 1 sighted students focus primarily on Hanyu Pinyin and basic orthographic knowledge (e.g., strokes, and radicals), justifying their exclusion from the present analyses.

After initial data collection, data screening was conducted prior to analysis. Valid data were retained for 101 blind students and 142 sighted students. Data screening followed predefined exclusion criteria. Specifically, three blind students withdrew from the testing session before completion and were therefore excluded from the dataset. In addition, three blind students were excluded due to excessive missing data, defined as more than 15% missing responses across the four key measures (compounding awareness, lexical inferencing, vocabulary knowledge, and Braille reading comprehension). For the sighted group, six students were excluded due to excessive missing data (>15%) in the lexical inferencing task.

Exclusion was due to incomplete data, failure to complete the task, invalid responses, or other predefined criteria. The blind students ranged in age from 9 to 15 years and were further categorized into the middle-grade (Grades 3–4; *n* = 44, *M* = 12.07, *SD* = 1.50) and upper-grade groups (Grades 5–6; *n* = 57, *M* = 12.79, *SD* = 1.15). Sighted students ranged in age from 6 to 11 years and were grouped into the lower-grade (Grade 2; *n* = 33, *M* = 7.88, *SD* = 0.49), middle-grade (Grades 3–4; *n* = 73, *M* = 9.40, *SD* = 0.70), and upper-grade groups (Grades 5–6; *n* = 36, *M* = 10.97, *SD* = 0.17). Students with blindness were older than sighted students at the same grade level, reflecting differences in schooling trajectories and timing of systematic Braille instruction. Students with blindness may enter school later, begin Braille learning at different ages, or experience more variable educational placements than do sighted students. Therefore, the comparison between students with blindness and sighted students in the present study should be interpreted as a grade-level comparison rather than an age-matched comparison. This pattern is also consistent with prior evidence that Braille readers often show delayed or more variable reading development relative to sighted readers (Savaiano et al., 2014; Xie et al., 2023). The descriptive characteristics of the sample are reported in Table 1 and Table 2.

Among the final sample of students with blindness, 44 were in the middle-grade group (Grades 3–4), and 57 were in the upper-grade group (Grades 5–6). Regarding regional distribution, in the middle-grade group, 5 students (11.4%) were from western regions, 9 (20.5%) were from central regions, and 30 (68.2%) were from eastern regions. In the upper-grade group, 12 students (21.1%) were from western regions, 8 (14.0%) from central regions, and 37 (64.9%) from eastern regions. Regarding the onset of blindness, 70 students (69.3%) were congenitally blind, while 31 students (30.7%) had acquired blindness, indicating that most participants experienced visual impairment from birth. With respect to Braille learning experience, in the middle-grade group, 26 students (59.1%) had 1–3 years of Braille learning experience, and 18 students (40.9%) had 4–6 years. In contrast, in the upper-grade group, only 3 students (5.3%) reported 1–3 years of experience, whereas 54 students (94.7%) reported 4–6 years of Braille learning, indicating longer and more sustained exposure in higher grades. In terms of educational background, 31 students (30.7%) with blindness had experience attending mainstream schools, whereas 70 students (69.3%) had no such experience. This distribution also varied by grade group: 19 students (43.2%) in the middle-grade group and 12 students (21.1%) in the upper-grade group had previously attended mainstream schools, while 25 (56.8%) and 45 (78.9%), respectively, had no such experience. Gender distribution was relatively balanced within groups, with 34 male students in each grade group; female students accounted for 10 students (22.7%) in the middle-grade group and 23 students (40.4%) in the upper-grade group.

### 2.2. Measures

#### 2.2.1. Compounding Awareness

The compounding production task was used to measure students’ compounding awareness. This measure has been used in studies of Chinese students with blindness (Chen et al., 2023; Xia et al., 2023; Xie et al., 2023). The task is designed to assess children’s ability to apply morphological knowledge, particularly compounding rules, to generate novel lexical items that are not part of their existing vocabulary.

The task was administered individually in an oral format. A trained researcher presented each item verbally by posing a question or describing a scenario, and participants were asked to produce a novel compound word that matched the intended meaning. Responses were independently scored by two trained graduate students using a 4-point scale (0–3), based on the extent to which the target morpheme was correctly extracted and the structural accuracy and conciseness of the response.

For example, in response to the prompt “用叶子做的盘子是什么？” (What do you call a plate made of leaves?), the target response “yè pán” (叶盘) received 3 points. Responses such as “yèzi pán” (叶子盘) or “yè pánzi” (叶盘子) received 2 points, whereas “yèzi pánzi” (叶子盘子) received 1 point. Responses that were structurally inappropriate, omitted the target morpheme, or were incorrect or absent received 0 points. In general, more concise and morphologically appropriate forms were assigned higher scores.

The test comprised 10 practice items and 25 scored items, with a maximum possible score of 75. Items were arranged in ascending order of difficulty, and testing was discontinued if a participant failed to respond correctly to five consecutive items. Previous studies with Chinese students with blindness have reported good internal consistency for this task (*Cronbach’s α* = 0.82–0.92) (Chen et al., 2023; Xia et al., 2023; Xie et al., 2023). In the present study, *Cronbach’s α* was 0.79, and inter-rater reliability was high (*r* = 0.96).

#### 2.2.2. Lexical Inferencing

The lexical inferencing task was operationalized as morpheme-based lexical inferencing of unfamiliar compound word meanings, which was taken from Cheng and Zhang (2023). It tested children’s ability to infer the meanings of morphologically complex words using morphemic cues. The present task did not provide contextual sentences. It was designed to focus on inference from morphemic structure, operationalizing lexical inference as students’ ability to infer the meanings of unfamiliar compound words from familiar constituent morphemes. Participants were presented with 16 bimorphemic words, each followed by four definition options. The internal structure of each compound provided cues to both grammatical category and semantic composition, and participants were required to select the most appropriate definition.

To minimize the influence of prior vocabulary knowledge, target words were selected based on two criteria. First, they did not appear in standard Chinese language textbooks, so neither blind nor sighted students were familiar with the words. Second, each compound consisted of two characters that were already familiar to the participants. This ensured that although the compound words themselves were likely novel, their meanings could be inferred through morphological analysis. The appropriateness of the target words was ensured through two objective procedures: (1) the use of a previously validated lexical inferencing instrument, and (2) verification against the national curriculum standards for both sighted and blind students. Experienced Chinese language teachers from schools for the blind were then invited to evaluate each of the 16 target words in terms of (1) curriculum relevance for students with blindness, (2) age-appropriateness for Grades 3–6, and (3) frequency and familiarity in Braille-based instructional materials.

For example, the unfamiliar compound word “lǚ fèi” (旅费, travel expenses) consists of “lǚ” (travel) and “fèi” (expense). The response options included: (a) a person who likes to travel everywhere, (b) the money one needs to spend on travel, (c) traveling is a waste of time, and (d) traveling with friends. The correct answer is (b). Successful performance requires identification of the constituent morphemes and application of the noun–noun compounding rule.

Previous research with Chinese sighted students has reported acceptable reliability for this task (*Cronbach’s α* = 0.78) (Cheng & Zhang, 2023). In the present study, internal consistency was acceptable (*Cronbach’s α* = 0.71) for the sample of 101 blind students.

#### 2.2.3. Vocabulary Knowledge

Vocabulary knowledge was assessed using a Vocabulary-Defining Task taken from Xie et al. (2023). This task evaluates the depth of lexical knowledge by requiring participants to provide explicit definitions of target words.

The task was administered individually in an oral format. Participants were presented with two-character Chinese words and asked to explain their meanings. For example, for the word “telephone,” a fully correct response would be: “A device that allows people who are far apart to communicate with each other.” Responses were independently scored by two trained graduate students using a 3-point scale: 2 points for a complete and accurate definition, 1 point for a partially correct response, and 0 points for an incorrect, vague, or irrelevant response.

The test consisted of one practice item and 37 scored items, yielding a maximum possible score of 74. Items were administered in a fixed order, and testing was discontinued if a participant failed to respond correctly to five consecutive items.

Previous research with Chinese blind students has demonstrated good reliability for this task (*Cronbach’s α* = 0.85) (Xie et al., 2023). In the present study, internal consistency was high (*Cronbach’s α* = 0.90), and inter-rater reliability was also strong (*r* = 0.91).

#### 2.2.4. Braille Reading Comprehension

Braille reading comprehension was assessed using a test taken from prior research by Chen et al. (2023), with reading materials drawn from the Progress in International Reading Literacy Study (PIRLS). Following expert review by experienced Chinese language teachers from schools for the blind, passages appropriate for middle- and upper-grade blind students were selected.

Participants completed two passages—one narrative and one expository. Each passage was followed by 10 multiple-choice comprehension questions. Each item included four response options. Students read the Braille texts tactually and recorded their responses on Braille paper. Each correct answer was awarded one point, yielding a maximum total score of 20.

Previous research with Chinese blind students in middle and upper primary grades has reported satisfactory internal consistency for this measure (*Cronbach’s α* = 0.86 and 0.87) (Chen et al., 2023). In the present study, internal consistency was acceptable, with *Cronbach’s α* coefficients of 0.73 for middle-grade students and 0.70 for upper-grade students.

All study materials were reviewed and refined by experts in special education, including researchers and experienced teachers from schools for the blind, to ensure their appropriateness for the target population.

### 2.3. Data Collection and Analysis

This study was approved by the Research Ethics Committee of the Faculty of Education, Beijing Normal University (IRB number: BNU202310100034, 10 November 2023), as also reported in the Institutional Review Board Statement. All procedures involving blind and sighted students were conducted in accordance with the ethical standards of the institutional review board and with the 1964 Helsinki Declaration and its later amendments. Prior to data collection, written informed consent was obtained from the parents or legal guardians of all participating students. Verbal assent was also obtained from each student before testing. Participation was voluntary, and participants were informed of their right to withdraw from the study at any time without penalty.

Background information was collected via questionnaires completed by homeroom teachers at schools for the blind. These questionnaires included information on students’ level of visual impairment, the presence of additional disabilities (e.g., intellectual disability), and other demographic characteristics.

All written materials were transcribed into Chinese Braille. Testing was administered by trained researchers who first provided standardized oral instructions. The compounding awareness and vocabulary knowledge tasks were conducted individually in quiet rooms, with two researchers independently scoring responses in real time. Lexical inferencing and Braille reading comprehension were administered in group settings by grade level, with students completing assessments in classrooms provided by the participating schools.

To reduce fatigue effects, each testing session lasted approximately 45 min. Data were collected from May to June 2024 and analyzed using SPSS 26.0. Descriptive statistics were first computed for all study variables. To address the first research question, which concerned how lexical inferencing performance in Chinese students with blindness compared with that of sighted students, descriptive statistics and group comparison analyses were conducted. To address the second, third, and fourth research questions, which concerned the predictive relations among lexical inferencing, compounding awareness, vocabulary knowledge, and Braille reading comprehension among students with blindness, hierarchical regression analyses were performed separately for middle-grade (Grades 3–4) and upper-grade (Grades 5–6) students with blindness.

## 3. Results

### 3.1. Normality Test

A one-sample Kolmogorov–Smirnov test indicated that compounding awareness, vocabulary knowledge, and Braille reading comprehension scores for students with blindness did not significantly deviate from normality (all *p* > 0.05). Lexical inferencing scores for students with blindness were normally distributed (*p* > 0.05), whereas lexical inferencing scores for sighted students significantly deviated from normality (*p* < 0.05).

### 3.2. Descriptive Statistics and Group Differences

Group differences in lexical inferencing were examined using Mann–Whitney U tests, comparing 101 students with blindness from eastern, central, and western China with 142 sighted students from Beijing (Table 3). The results indicated that students with blindness scored significantly lower than their sighted peers at both middle and upper primary grade levels (*Z* = −6.44, *p* < 0.001, *r* = 0.60; *Z* = −6.31, *p* < 0.001, *r* = 0.65).

Gender-stratified analyses revealed a consistent pattern: male students with blindness scored significantly lower than sighted males (*Z* = −5.24, *p* < 0.001, *r* = 0.44), and female students with blindness likewise scored significantly lower than sighted females (*Z* = −4.88, *p* < 0.001, *r* = 0.49). Overall, lexical inferencing performance was significantly lower among students with blindness, regardless of gender or grade, suggesting a robust effect of visual status.

To further contextualize these differences, cross-grade comparisons were conducted. Middle-grade students with blindness scored significantly lower than lower-grade sighted students (Z = −3.70, *p* < 0.001, *r* = 0.42). Upper-grade students with blindness scored significantly lower than middle-grade sighted peers (*Z* = −3.99, *p* < 0.001, *r* = 0.35), but did not differ significantly from lower-grade sighted peers (*Z* = −0.72, *p* > 0.05, *r* = 0.08). Although lexical inferencing improved with grade level in both groups, students with blindness consistently lagged behind their sighted peers, with a substantial and persistent performance gap.

Descriptive statistics and group differences were examined for four variables among students with blindness in middle and upper primary grades: Braille reading comprehension, lexical inferencing, compounding awareness, and vocabulary knowledge (Table 4).

Results indicated that upper-grade students significantly outperformed middle-grade students in Braille reading comprehension (*M* = 11.21 vs. 9.50, *t* = −2.54, *p* < 0.05, *Hedges’ g* = −0.51), lexical inferencing (*M* = 11.81 vs. 9.61, *t* = −3.68, *p* < 0.001, *Hedges’ g* = −0.73), compounding awareness (*M* = 47.93 vs. 38.75, *t* = −4.09, *p* < 0.001, *Hedges’ g* = −0.81), and vocabulary knowledge (*M* = 42.07 vs. 35.55, *t* = −3.78, *p* < 0.001, *Hedges’ g* = −0.75).

In addition, students with longer durations of Braille learning demonstrated significantly higher lexical inferencing (*M* = 11.26 vs. 9.83, *t* = −2.11, *p* < 0.05, *Hedges’ g* = −0.46) and compounding awareness (*M* = 45.44 vs. 40.17, *t* = −2.02, *p* < 0.05, *Hedges’ g* = −0.44) than those with shorter learning experience.

Descriptive statistics and group differences were examined for four variables—Braille reading comprehension, lexical inferencing, compounding awareness, and vocabulary knowledge—among middle- and upper-grade primary students with blindness (Table 5). Among middle-grade students, no significant differences were observed across gender, prior mainstream schooling experience, duration of Braille learning, or age of blindness onset (*p* > 0.05).

In contrast, among upper-grade students, those with prior mainstream schooling experience demonstrated significantly higher vocabulary knowledge (*M* = 46.83) than those without such experience (*M* = 40.80, *t* = −2.17, *p* < 0.05, *Hedges’ g* = −0.70). No other group differences reached statistical significance (*p* > 0.05).

### 3.3. Correlation Analysis

Table 6 presents the bivariate correlations among compounding awareness, lexical inferencing, vocabulary knowledge, and Braille reading comprehension for 44 middle-grade and 57 upper-grade blind students. Across both grade groups, compounding awareness was positively and significantly associated with braille reading comprehension, lexical inferencing, and vocabulary knowledge (all *p* < 0.001).

Vocabulary knowledge also showed significant positive correlations with both lexical inferencing and Braille reading comprehension (both *p* < 0.001), and lexical inferencing was, in turn, significantly correlated with Braille reading comprehension (*p* < 0.001).

In addition, within the upper-grade group, region was significantly associated with compounding awareness (*p* < 0.05), and prior mainstream schooling experience was significantly related to vocabulary knowledge (*p* < 0.05).

### 3.4. Hierarchical Regression Analysis

Drawing on prior research and the results of the correlation analyses, four variables—compounding awareness, lexical inferencing, vocabulary knowledge, and Braille reading comprehension—were entered into a series of multiple linear regression models.

In Grades 3 to 4 (Table 7), blind students’ compounding awareness explained variance in Braille reading comprehension (Δ*R*^2^ = 0.37, *t* = 4.99, *p* < 0.001) as well as in vocabulary knowledge (Δ*R*^2^ = 0.33, *t* = 4.54, *p* < 0.001). Meanwhile, blind students’ lexical inferencing significantly explained variance in Braille reading comprehension (Δ*R*^2^ = 0.45, *t* = 10.11, *p* < 0.001) and vocabulary knowledge (Δ*R*^2^ = 0.37, *t* = 7.02, *p* < 0.001). Compounding awareness explained variance in lexical inferencing (Δ*R*^2^ = 0.22, *t* = 3.40, *p* < 0.01). Vocabulary knowledge also explained variance in Braille reading comprehension (Δ*R*^2^ = 0.05, *t* = 4.16, *p* < 0.001). These results suggest that both compounding awareness and lexical inferencing play a role in the vocabulary knowledge and Braille reading comprehension during Grades 3 to 4 of elementary school.

In Grades 5 to 6 (Table 8), as in Grades 3 to 4, compounding awareness explained variance in lexical inferencing (Δ*R*^2^ = 0.21, *t* = 3.87, *p* < 0.001). However, when lexical inferencing was entered in Equations 1 and 2, compounding awareness did not explain variance in Braille reading comprehension and vocabulary knowledge. Blind students’ lexical inferencing explained variance in Braille reading comprehension (Δ*R*^2^ = 0.41, *t* = 7.56, *p* < 0.001) and vocabulary knowledge (Δ*R*^2^ = 0.38, *t* = 8.28, *p* < 0.001) after prior mainstream schooling experience was controlled. These results suggest that lexical inferencing plays a unique role in vocabulary knowledge and Braille reading comprehension during late elementary school (Grades 5 to 6).

## 4. Discussion

The present study examined lexical inferencing in Chinese students with blindness and tested how compounding awareness, lexical inferencing, and vocabulary knowledge were associated with Braille reading comprehension in the middle and upper primary grades. The findings answered the four research questions in a relatively consistent pattern. First, students with blindness showed lower lexical inferencing performance than sighted students in both Grades 3 to 4 and Grades 5 to 6, although lexical inferencing improved with grade level. Second, lexical inferencing significantly predicted both vocabulary knowledge and Braille reading comprehension among students with blindness. Third, compounding awareness significantly predicted lexical inferencing in both Grades 3 to 4 and Grades 5 to 6 students. Fourth, the relative role of compounding awareness and lexical inferencing differed by grade group.

### 4.1. Comparison of Lexical Inferencing Between Chinese Primary School Students with Blindness and Sighted Peers

The present study found that lexical inferencing improved with grade level among both Chinese students with blindness and their sighted peers. Metalinguistic awareness has been shown to be closely associated with lexical inferencing (Cheng & Zhang, 2023). As language development advances, metalinguistic awareness becomes increasingly refined, enabling learners to integrate morphological knowledge more effectively when inferring the meanings of unfamiliar words. In addition, as tactile reading skills develop through extensive reading and language practice, students with blindness gain access to a broader range of lexical and syntactic information, which may further facilitate the gradual improvement of lexical inferencing ability.

Despite these gains, students with blindness in Grades 3 to 4 and Grades 5 to 6 continued to perform significantly below their sighted peers and even below younger sighted students. From a language development perspective, this persistent gap may reflect differences in the developmental trajectory of underlying metalinguistic skills. Chen et al. (2023) reported that Chinese students with blindness scored significantly lower than their sighted peers on compounding awareness in both Grades 3 to 4 and Grades 5 to 6, and that this gap became more pronounced with increasing grade level. Given that compounding awareness provides an important foundation for lexical inferencing, its slower development among students with blindness may contribute to the widening disparity in lexical inferencing observed between the two groups. This pattern also suggests that visual experience plays an important role in lexical inferencing. Visual–linguistic dual coding facilitates conceptual understanding and lexical processing (Paivio, 2008), whereas the absence of visual input may constrain the development of rich lexical representations and semantic networks in students with blindness (Landau & Gleitman, 1985).

The delayed development of lexical inferencing may also be related to characteristics of Chinese Braille. Braille reading is generally less efficient for information extraction (Emerson et al., 2009), and Chinese Braille is further characterized by a high prevalence of homophones and strong phonological–orthographic consistency (Chen et al., 2023). Consequently, students with blindness may require greater memory and inferential effort during reading, making them more vulnerable to cognitive load in tasks requiring the integration of multiple sources of information.

### 4.2. Effects of Grade Level, Years of Braille Learning, and Prior Mainstream Schooling Experience on Chinese Primary School Students with Blindness

The present study found that Braille reading comprehension, compounding awareness, and vocabulary knowledge among Chinese primary school students with blindness improved with grade level. Students with longer Braille learning experience also demonstrated significantly higher levels of compounding awareness, consistent with previous research (Chen et al., 2023; Xie et al., 2023). Prior studies suggest that increased exposure to complex texts facilitates more rapid and accurate processing of morphemic information (Wang & Zhang, 2021; Ku & Anderson, 2003). As tactile Braille reading proficiency develops, students gain access to a wider range of texts, which may strengthen their understanding of morphemes and compounding structures, thereby promoting the development of compounding awareness (Kuo & Anderson, 2006).

The present study further found that in Grades 5 to 6, students with prior mainstream schooling experience demonstrated significantly higher levels of vocabulary knowledge. This finding suggests that mainstream school experience may facilitate the cumulative development of reading-related abilities among students with blindness. Compared with specialized schools for the blind, mainstream schools generally provide richer language input, greater lexical diversity in classroom instruction, and more opportunities for peer interaction. Through increased exposure to contextualized language use and authentic social communication, students with blindness may develop more refined semantic representations, with these advantages becoming more evident at higher grade levels.

### 4.3. Relationships Among Compounding Awareness, Lexical Inferencing, Vocabulary Knowledge, and Braille Reading Comprehension

The present study found that lexical inferencing explained variance in vocabulary knowledge and Braille reading comprehension among middle- and upper-grade blind students. According to the Reading Systems Framework (Perfetti & Stafura, 2014), lexical inferencing is closely related to vocabulary knowledge during the integration process from word recognition to text comprehension. In the present study, lexical inferencing reflects an individual’s understanding of linguistic rules and semantic transfer. Blind students with stronger lexical inferencing may demonstrate greater morphological sensitivity, enabling them to efficiently recognize Braille symbol patterns and associate them with previously acquired morphemic knowledge. This process helps them understand how identical Braille symbols may vary across different combinations and contexts, thereby enhancing unfamiliar word comprehension. For example, when identifying the Braille word “
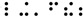
” (“旅费”, travel expenses), students who can recognize the morphemic components “
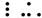
” (“旅”, travel) and “
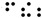
” (“费”, expense) and integrate their meanings may infer that the word refers to expenses incurred during travel. Moreover, stronger lexical inferencing abilities may help blind students process unfamiliar words more efficiently during Braille reading, facilitating information integration and improving comprehension of complex sentences and passages without frequent disruptions caused by unfamiliar vocabulary.

The present study found that compounding awareness was a significant predictor of lexical inferencing among both middle- and upper-grade blind students. This finding is consistent with previous studies on Chinese print reading among sighted children.

From a language development perspective, in earlier stages, children rely primarily on direct word-level learning, with limited ability to analyze internal word structure. As development progresses, metalinguistic awareness—particularly morphological awareness such as compounding awareness—becomes increasingly important, enabling learners to decompose words into meaningful morphemic units and to use structural knowledge to infer the meanings of unfamiliar lexical items.

From a language-specific perspective, Chinese is characterized by a high proportion of compound words, in which the meanings of whole words are often transparently related to the meanings of their constituent morphemes, thereby facilitating the use of morphemic information in word learning and inference. According to morphemic decomposition theory, when encountering complex or unfamiliar words, learners can rely on compounding awareness to segment and integrate constituent morphemes, thereby constructing word meanings through inferential processing.

Similarly, Chinese Braille contains many compound words, and blind students learn Braille word segmentation and connected-writing rules from the early stages of Braille learning. As a result, blind students with stronger compounding awareness may recognize Braille symbol patterns more efficiently, analyze word structures, and infer unfamiliar word meanings during Braille reading. For example, when reading the Braille word “
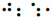
” (“日食”, solar eclipse), students who understand the meanings of the morphemes “日” (sun) and “食” (eat) may infer that the word relates to an astronomical phenomenon involving the sun. Combined with prior knowledge, they may further infer the meaning of a “solar eclipse.”

Finally, it should be noted that the compounding awareness and lexical inferencing were closely related, which may partly reflect the fact that the lexical inferencing task involved unfamiliar compound words whose meanings could be inferred from familiar constituent morphemes. Thus, although the task assessed unfamiliar-word meaning inference, it also included morphological processing components that overlap with those involved in compounding awareness. This should be considered when interpreting the predictive relationship between the two variables.

### 4.4. Differential Contributions of Compounding Awareness and Lexical Inferencing

Hierarchical regression analyses further suggested potential grade-level differences in the contributions of compounding awareness and lexical inferencing to vocabulary knowledge and Braille reading comprehension among blind students. Specifically, compounding awareness and lexical inferencing jointly contributed to vocabulary knowledge and Braille reading comprehension among middle-grade blind students, whereas lexical inferencing played a more prominent role among upper-grade blind students. This may be associated with the developmental characteristics of compounding awareness across different stages of reading development in blind students.

According to the stages of reading development theory proposed by Chall (1983), Braille reading development is similar to print reading development in sighted children with respect to the developmental stages involved (Steinman et al., 2006). During the “learning to read” stage, blind students typically rely more on decoding skills based on metalinguistic awareness (Wormsley & D’Andrea, 1997). As children progress through elementary school, the development of compounding awareness gradually plateaus with increasing age and grade level (L. Zhang et al., 2023). At the same time, improvements in braille tactile reading ability and cognitive development enable blind students to move from “learning to read” to “reading to learn.”

At this stage, more cognitive resources are allocated to processing and analyzing unfamiliar words. This process involves not only relatively basic processes, such as retrieval, but also higher-order cognitive skills, including inferential integration, which gradually becomes more important for word meaning construction (Zhao et al., 2024). Consequently, the role of compounding awareness may weaken or even become non-significant. In addition, the dynamic relations hypothesis of the direct and indirect effects model of reading suggests that foundational reading-related skills, including metalinguistic awareness, may play different roles across stages of reading development, reflecting the dynamic and complex nature of reading development (Kim, 2020). Consistent with this view, Xia et al. (2023) found that compounding awareness significantly predicted Braille sentence and passage comprehension among Chinese blind students in Grades 3–4, whereas this predictive effect was no longer significant among students in Grades 5–6.

## 5. Implications for Practice

The findings offer cautious practical implications for schools for the blind. First, the results highlight the potential importance of enriched language environments and early instruction. Students with prior mainstream schooling experience demonstrated stronger vocabulary knowledge, raising the possibility that high-quality language input and interactive learning contexts may be beneficial for vocabulary development. The findings may reflect, in part, the possibility that early and systematic Braille instruction supports the development of foundational reading skills and strategic reading processes. Second, compounding awareness appears to play a particularly important role during the middle primary grades. Future instructional work may explore activities that encourage students to analyze and manipulate the morphemic structure of compound words, such as morpheme segmentation and recombination tasks based on familiar everyday contexts. Auditory activities that highlight morphemic boundaries during Braille reading, as well as the use of Braille cards containing individual morphemes to construct new compound words, may be examined as potential ways to foster morphological awareness. However, the effectiveness of these instructional approaches remains to be established through intervention studies. Third, middle- and upper-grade students with blindness demonstrated weaker morphology-based lexical inferencing performance than their sighted peers. This finding may indicate that explicit instruction targeting lexical inferencing warrants further empirical examination. For example, teachers’ use of morpheme-based instruction, including guiding students to infer the meanings of unfamiliar words through constituent morpheme analysis and encouraging them to combine familiar morphemes to generate novel compound words and sentences, may be a promising instructional approach that warrants further investigation in future research. Although these activities are theoretically motivated by the present findings, future longitudinal and intervention-based studies are needed to determine whether such instructional practices effectively improve lexical inferencing, vocabulary knowledge, and Braille reading comprehension.

## 6. Limitations and Future Directions

Despite its contributions, this study has several limitations. First, group comparisons may be influenced by differences in educational context. A limitation of this study is that the comparison between the two groups should be interpreted with caution because the students with blindness and sighted students differed not only in visual status but also in educational placement, schooling trajectories, and learning environments. Future research should minimize confounding effects of the educational context by using matched sampling or more comparable schooling environments when comparing students with blindness and sighted students. Such designs would help disentangle the effects of visual status from differences in educational placement and learning experience.

Second, the sample sizes within grade-level subgroups were relatively small, which may limit the statistical power of subgroup analyses and the generalizability of the findings. Moreover, mediation analyses could not be conducted. Future studies with larger and more balanced samples are needed to further validate these results. Longitudinal studies with larger samples are needed to investigate developmental trajectories and evaluate the effectiveness of interventions targeting morphological awareness and lexical inferencing across grade levels.

Third, the present study specifically focused on students with blindness and did not include participants with low vision or other levels of visual impairment. Accordingly, the findings should not be generalized to the broader spectrum of visual impairment without further empirical evidence. In addition, although information on the onset of blindness (congenital versus acquired) was collected, no significant differences were found between these subgroups, and this factor was not included in the main analyses. Nevertheless, future research should recruit participants representing the full spectrum of visual impairment, as defined by ICD-11 (e.g., low vision, moderate, and severe visual impairment, and blindness), as well as both congenital and acquired blindness. Such work would facilitate comparisons across internationally recognized classifications of visual impairment and provide a more comprehensive understanding of how visual impairment severity and onset shape language and literacy development.

Fourth, other aspects of Braille experience, including age of Braille acquisition, age of blindness onset, and reading frequency, were not included in the present dataset. These variables were not collected because they could not be obtained with sufficient precision: during data collection, both students and teachers were only able to provide approximate estimates, and many students were unable to accurately report the timing of vision loss or Braille acquisition. Given this lack of reliable retrospective information, these variables were not suitable for inclusion in the current analyses. Therefore, observed associations should be interpreted with caution, as they may partly reflect unmeasured differences in reading exposure. Future studies should systematically incorporate multidimensional indices of Braille experience.

Finally, the assessment of lexical inferencing was limited in ecological validity. The task focused on morpheme-based inference of compound word meanings without sentence- or discourse-level contexts, capturing only a restricted form of inferential processing. Future research should therefore employ more ecologically valid instruments embedded in authentic reading contexts.

## 7. Conclusions

Results showed that (1) students with blindness performed lower than sighted peers on lexical inferencing despite grade-related improvement; (2) lexical inferencing significantly predicted both vocabulary knowledge and Braille reading comprehension in both middle-grade students and upper-grade students, whereas compounding awareness significantly predicted lexical inferencing; (3) the relative role of compounding awareness and lexical inferencing differed by grade group. In middle-grade students, both compounding awareness and lexical inferencing predicted vocabulary knowledge and Braille reading comprehension, with vocabulary knowledge also predicting reading comprehension. In upper-grade students, lexical inferencing remained a significant predictor of both vocabulary knowledge and Braille reading comprehension, whereas compounding awareness no longer directly predicted either outcome.

## Figures and Tables

**Table 1:** Basic information table for blind students.

Middle-Grade Blind Students (Grades 3–4) (*n* = 44)		*n* (%)	Upper-Grade Blind Students (Grades 5–6) (*n* = 57)		*n* (%)
Region	Western	5 (11.4%)	Region	Western	12 (21.1%)
	Central	9 (20.5%)		Central	8 (14.0%)
	Eastern	30 (68.2%)		Eastern	37 (64.9%)
Gender	Male	34 (77.3%)	Gender	Male	34 (59.6%)
	Female	10 (22.7%)		Female	23 (40.4%)
**Prior Mainstream Schooling Experience**	Yes	19 (43.2%)	**Prior Mainstream Schooling Experience**	Yes	12 (21.1%)
	No	25 (56.8%)		No	45 (78.9%)
Years of Braille learning	1–3 years	26 (59.1%)	Years of Braille learning	1–3 years	3 (5.3%)
	4–6 years	18 (40.9%)		4–6 years	54 (94.7%)
Onset of blindness	Congenital	32 (72.7%)	Onset of blindness	Congenital	38 (66.7%)
	Acquired	12 (27.3%)		Acquired	19 (33.3%)

**Table 2:** Basic information for sighted students.

Sighted Students in the Beijing Area (*n* = 142)		*n* (%)
Gender	Male	77 (54.2%)
	Female	65 (45.8%)
Grade Level	Lower Grade (Grade 2)	33 (23.3%)
	Middle Grade (Grades 3–4)	73 (51.4%)
	Upper Grade (Grades 5–6)	36 (25.4%)

**Table 3:** Comparison of lexical inferencing between blind and sighted students.

Blind Students vs. Sighted Students		*n*	*M* ± *SD*	*Z*-Value	*r*
Middle-grade	Blind	44	9.61 ± 3.16	−6.44 ***	0.60
	Sighted	73	13.64 ± 2.45		
Upper-grade	Blind	57	11.81 ± 2.82	−6.31 ***	0.65
	Sighted	36	15.22 ± 0.96		
Middle-grade Blind		44	9.61 ± 3.16	−3.70 ***	0.42
Lower-grade Sighted		33	12.24 ± 2.83		
Upper-grade Blind		57	11.81 ± 2.82	−0.72	0.08
Lower-grade Sighted		33	12.24 ± 2.83		
Upper-grade Blind		57	11.81 ± 2.82	−3.99 ***	0.35
Middle-grade Sighted		73	13.64 ± 2.45		
Male	Blind	68	10.85 ± 3.16	−5.24 ***	0.44
	Sighted	77	13.44 ± 2.68		
Female	Blind	33	10.85 ± 3.18	−4.88 ***	0.49
	Sighted	65	14.05 ± 2.22		

**Note:** *M* = mean, *SD* = standard deviation, *** *p* < 0.001, two-tailed.

**Table 4:** Descriptive statistics and group differences in study variables among middle- and upper-grade blind students.

Variable	Grade Level	*n*	*M* ± *SD*	*t*-Value	*Hedges’ g*	Years of Braille Learning	*n*	*M* ± *SD*	*t*-Value	*Hedges’ g*
Braille Reading Comprehension	Middle Grade	44	9.50 ± 3.11	−2.54 *	−0.51	1–3 years	29	10.00 ± 3.39	−0.86	−0.19
	Upper Grade	57	11.21 ± 3.53			4–6 years	72	10.65 ± 3.47		
Lexical Inferencing	Middle Grade	44	9.61 ± 3.16	−3.68 ***	−0.73	1–3 years	29	9.83 ± 3.11	−2.11 *	−0.46
	Upper Grade	57	11.81 ± 2.82			4–6 years	72	11.26 ± 3.10		
Compounding Awareness	Middle Grade	44	38.75 ± 10.81	−4.09 ***	−0.81	1–3 years	29	40.17 ± 11.71	−2.02 *	−0.44
	Upper Grade	57	47.93 ± 11.49			4–6 years	72	45.44 ± 11.92		
Vocabulary Knowledge	Middle Grade	44	35.55 ± 8.32	−3.78 ***	−0.75	1–3 years	29	38.43 ± 10.36	−0.55	−0.12
	Upper Grade	57	42.07 ± 8.83			4–6 years	72	39.55 ± 8.70		

**Note:** *M* = mean; *SD* = standard deviation, * *p* < 0.05, *** *p* < 0.001, two-tailed.

**Table 5:** Descriptive statistics and group differences in study variables among upper-grade blind students.

Variable	Prior Mainstream Schooling Experience	*n*	*M* ± *SD*	*t*-Value	*Hedges’ g*
Braille Reading Comprehension	No	45	10.89 ± 3.21	−1.34	−0.43
	Yes	12	12.42 ± 4.50		
Lexical Inferencing	No	45	11.60 ± 2.71	−1.07	−0.34
	Yes	12	12.58 ± 3.23		
Compounding Awareness	No	45	48.71 ± 10.95	0.99	0.32
	Yes	12	45.00 ± 13.43		
Vocabulary Knowledge	No	45	40.80 ± 7.25	−2.17 *	−0.70
	Yes	12	46.83 ± 12.45		

**Note:** *M* = mean, *SD* = standard deviation, * *p* < 0.05, two-tailed.

**Table 6:** Correlations among variables in middle- and upper-grade blind students.

Grade Level	Variable	1	2	3	4
Middle Grade (*n* = 44)	1 Braille Reading Comprehension	-			
	2 Lexical Inferencing	0.88 ***	-		
	3 Compounding Awareness	0.61 ***	0.47 **	-	
	4 Vocabulary Knowledge	0.88 ***	0.80 ***	0.57 ***	-
	Region	0.17	0.09	0.13	−0.01
	Age	−0.10	−0.01	−0.12	−0.03
	Gender	−0.23	−0.18	−0.10	−0.12
	Years of Braille Learning	−0.11	0.04	−0.07	−0.20
	Prior Mainstream Schooling Experience	0.05	0.09	−0.07	0.27
	Age of Blindness Onset	0.02	0.13	0.00	0.03
Upper Grade (*n* = 57)	1 Braille Reading Comprehension	-			
	2 Lexical Inferencing	0.78 ***	-		
	3 Compounding Awareness	0.46 ***	0.46 ***	-	
	4 Vocabulary Knowledge	0.71 ***	0.81 ***	0.45 ***	-
	Region	0.23	0.12	0.29 *	0.20
	Age	0.10	0.12	−0.007	0.09
	Gender	−0.02	0.006	0.03	−0.05
	Years of Braille Learning	−0.05	−0.07	0.02	−0.26
	Prior Mainstream Schooling Experience	0.18	0.14	−0.13	0.28 *
	Age of Blindness Onset	0.05	0.02	0.04	0.13

**Note:** * *p* < 0.05, ** *p* < 0.01, *** *p* < 0.001, two-tailed.

**Table 7:** Multiple linear regression testing of study variables in Grades 3 to 4 blind students (*n* = 44).

	Outcome Variables	Step	Predictors	*β*	95% *CI*	*VIF*	*t*-Value	*R* ^2^	Δ*R*^2^	*F*
**Equation (1)**	Braille Reading Comprehension	1	Compounding Awareness	0.61	[0.10, 0.25]	1.00	4.99 ***	0.37	0.37	24.88 ***
		2	Compounding Awareness	0.26	[0.03, 0.12]	1.28	3.46 ***	0.82	0.45	93.55 ***
			Lexical Inferencing	0.76	[0.60, 0.90]	1.28	10.11 ***			
		3	Compounding Awareness	0.15	[0.004, 0.08]	1.49	2.20 *	0.87	0.05	92.87 ***
			Lexical Inferencing	0.47	[0.27, 0.65]	2.81	4.98 ***			
			Vocabulary Knowledge	0.42	[0.08, 0.23]	3.28	4.16 ***			
**Equation (2)**	Vocabulary Knowledge	1	Compounding Awareness	0.57	[0.25, 0.64]	1.00	4.54 ***	0.33	0.33	20.57 ***
		2	Compounding Awareness	0.26	[0.05, 0.35]	1.28	2.63 *	0.70	0.37	46.79 ***
			Lexical Inferencing	0.68	[1.29, 2.32]	1.28	7.02 ***			
**Equation (3)**	Lexical Inferencing	1	Compounding Awareness	0.47	[0.06, 0.22]	1.00	3.40 **	0.22	0.22	11.57 **

**Note:** * *p* < 0.05, ** *p* < 0.01, *** *p* < 0.001, two-tailed. Equation (1), Equation (2) and Equation (3) refer to three hierarchical regression formula in this study.

**Table 8:** Multiple linear regression testing of study variables in Grades 5 to 6 blind students (*n* = 57).

	Outcome Variables	Step	Predictors	*β*	95% *CI*	*VIF*	*t*-Value	*R* ^2^	Δ*R*^2^	*F*
**Equation (1)**	Braille Reading Comprehension	1	Compounding Awareness	0.46	[0.07, 0.22]	1.00	3.82 ***	0.21	0.21	14.62 ***
		2	Compounding Awareness	0.13	[−0.02, 0.10]	1.27	1.33	0.62	0.41	43.30 ***
			Lexical Inferencing	0.72	[0.66, 1.14]	1.27	7.56 ***			
**Equation (2)**	Vocabulary Knowledge	1	Experience in regular schools	0.28	[0.47, 11.6]	1.00	2.17 *	0.08	0.08	4.72 *
		2	Experience in regular schools	0.35	[2.59, 12.32]	1.02	3.07 **	0.32	0.24	12.83 ***
			Compounding Awareness	0.50	[0.21, 0.56]	1.02	4.40 ***			
		3	Experience in regular schools	0.20	[0.92, 7.58]	1.08	2.55 *	0.70	0.38	42.09 ***
			Compounding Awareness	0.15	[−0.02, 0.25]	1.34	1.69			
			Lexical Inferencing	0.72	[1.70, 2.78]	1.35	8.28 ***			
**Equation (3)**	Lexical Inferencing	1	Compounding Awareness	0.46	[0.06, 0.17]	1.00	3.87 ***	0.21	0.21	14.95 ***

**Note:** * *p* < 0.05, ** *p* < 0.01, *** *p* < 0.001, two-tailed. Equation (1), Equation (2) and Equation (3) refer to three hierarchical regression formula in this study.

## Data Availability

The data are currently not publicly available due to participant privacy, but they are available from the corresponding author upon reasonable request.
